# Effectiveness of transverse tibial bone transport in treatment of diabetic foot ulcer: A systematic review and meta-analysis

**DOI:** 10.3389/fendo.2022.1095361

**Published:** 2023-01-04

**Authors:** Xing-xi Hu, Zheng-zhong Xiu, Gui-chun Li, Ji-yuan Zhang, Long-jun Shu, Zhong Chen, Han Li, Qing-feng Zou, Quan Zhou

**Affiliations:** ^1^ Department of Orthopedics and Trauma, The Affiliated Hospital of Yunnan University (The Second People’s Hospital of Yunnan Province, The Eye Hospital of Yunnan Province), Kunming, Yunnan, China; ^2^ Department of Orthopedics, The First People’s Hospital of Dali City, Dali, China; ^3^ Western Yunnan University of Applied Science and Technology, Dali, China

**Keywords:** transverse tibial bone transport, meta-analysis, ulceration healing, neovascularization, diabetic foot ulceration

## Abstract

**Background:**

Diabetic foot ulcerations (DFUs) are a common but highly morbid complication of long-standing diabetes, carrying high rates of associated major amputation and mortality. Transverse tibial bone transport (TTT) has recently been applied for treatment of DFUs with the aim of accelerating wound healing. This study was performed to evaluate the effectiveness and safety of TTT in patients with DFUs.

**Methods:**

Two authors independently retrieved the platforms of PubMed, Embase and CENTRAL, to identify studies associated with treatment of DFUs with TTT. Quantitative meta-analyses were performed to pool all available outcomes about the effectiveness and complications of TTT operation, with fixed- (I^2^<50%) or random-effect (I^2^>50%) model according to I^2^.

**Results:**

A total of 7 studies, involving 818 participants, were included, with 661 participants treated with TTT operation. The pooled healing rate and limb salvage rate were 0.96 (95%CI: 0.93~0.98) and 0.98 (95%CI: 0.95~1.00) respectively after treatment with TTT. The pooled mean healing time was 15.03 (95%CI: 9.05~21.00) months. When compared with the pre-operative baseline values, the ankle-brachial index (ABI, MD: 0.23; 95%CI: 0.03~0.44; p<0.001), skin temperature (MD: 1.56; 95%CI: 0.30~2.81; p<0.001), and visual analogue scale (VAS, MD: 3.70; 95%CI: 1.97~5.44; p<0.001) were significantly improved at the final follow-up. When compared with non-TTT group, the TTT group was associated with higher healing rate (OR: 10.43; 95%CI: 3.96~27.43; p<0.001) and limb salvage rate (OR: 9.65; 95%CI: 3.30~28.20; p<0.001). Concerning the complications of the TTT process, the pooled risks of fracture at transportation site and pin-site infection were 0.02 (95%CI: 0.00~0.04) and 0.08 (95%CI: 0.00~0.22), respectively; and the DFU recurrence rate in TTT group was significantly lowered comparing to that of the non-TTT group (RR: 0.18; 95%CI: 0.06~0.49; p=0.001).

**Conclusions:**

TTT operation was associated with high healing rate and limb salvage rate, and could significantly improve the ABI, skin temperature, and VAS after operation. When compared with the control group, TTT group provided significantly higher healing rate and limb salvage rate. However, TTT operation should be conducted with caution concerning the incidences of fracture at tibia, infection at pin channels and necrosis of skin overlying the anterior tibia.

## Introduction

Diabetes has gradually emerged as one of the most globally challenging chronic diseases, its prevalence has increased significantly over the past few decades, resulting in disabling and costly complications, life-threatening conditions, and reducing life expectancy (1, 2). The IDF (International Diabetes Federation) Diabetes Atlas, 10th edition, showed that by 2021, 1 in 10 adults worldwide will have diabetes and the number of people with diabetes will continue to increase rapidly in the future (1). Diabetic foot is an infection, ulceration and deep tissue destruction of the foot caused by neuropathy and vascular disease of the lower limbs in diabetic patients (3). About 19% - 34% of people with diabetes are likely to have diabetic foot ulcers (DFUs) in their lifetime (4, 5). DFU is a common but severely prevalent complication of long-term diabetes with high rates of associated amputation and mortality (2, 4, 6, 7). The global burden of DFUs is steadily increasing as the global prevalence of diabetes rises (8). It is well known that the outcomes of diabetes and DFUs depend heavily on the social determinants of health, with worse outcomes for ethnic minorities and socio-economically disadvantaged groups (2).

Tibial bone transverse transport (TTT) is an extension of the Ilizarov technique (9). Being different from the longitudinal transport of the osteotomy segment according to the Ilizarov external fixation technique, this procedure involves transverse traction of the tibial osteotomy segment. The primary goal of TTT is not osteogenesis, but local vascular tissue regeneration (10, 11). Originated in the law of “stress-tension”, continuous distraction of the tibial cortex promotes cellular metabolism, accelerates tissue regeneration, reestablishes microcirculation and restores blood oxygen to the lower limbs. This technique is mainly used for the treatment of chronic ischaemic diseases of the lower extremities, at this stage (12).

China’s Qu et al. (13) firstly applied TTT to clinical practice in China, which not only introduced TTT to China, but also initiated the exploration of TTT among Chinese scholars. Ou et al. (3) found that TTT can improve blood circulation in the affected limb, promote wound healing in diabetic feet, reduce amputation rates, and significantly increase the expression of early serum angiogenic factors, which may contribute to the mechanism of accelerated healing of diabetic foot wounds. Additionally, Nie et al. (14) found that TTT is an effective treatment for refractory non-diabetic lower extremity ulcers compared to conventional surgery.

To our knowledge, TTT has been used many times in recent years to treat diabetic foot. With the continuous development of orthopaedic procedures, the use of TTT for diabetic foot has become more and more mature, and several clinical studies have shown that this method has significant efficacy in treating diabetic foot with less adverse effects (15–18), but there is still no relevant publication on evidence-based rationale. Accordingly, we conducted this quantitative meta-analysis to thoroughly evaluate the clinical efficacy and safety of TTT in the treatment protocols of DFUs.

## Materials and methods

This systematic review and meta-analysis was performed according to the guideline outlined in Preferred Reporting Items for Systematic Reviews and Meta-analysis (PRISMA) statement. The PRISMA checklist is presented in **Appendix 1**.

### Data source and study searching

Two authors retrieved the electronic databases, including PubMed, Embase and the Cochrane Central Register of Controlled Trials (CENTRAL) from the inception dates to Nov. 2022, independently. The keywords used for study searching include “diabetic foot ulcer”, “diabetic foot”, “transverse tibial bone transport”, and so on. We combined the subject terms and free terms together to ensure full coverage of the potential eligible studies. The list of the searching strategies of three databases is available in **Appendix 2**. The related studies in the references list of each included study were also hand-searched and included for analysis.

### Inclusion and exclusion criteria

After exporting the literature records from the databases, two authors screened all of them one by one to identify eligible studies, according to the inclusion criteria as follows: (1) patients were diagnosed with DFU (type-I or -II diabetes)DFU; (2) patients were operated with TTT on the DFU affected leg; (3) studies observed the treatment outcomes of the TTT surgery, such as healing time, healing rate, ABI, skin temperature, VAS pain scale, complications, and so on; (4) clinical studies designed as randomized controlled trials (RCTs), cohort studies, case-control studies, or case series. The publication language was restricted in English.

Studies would be excluded when meeting the following criteria: (1) duplicated studies; (2) patients operated for foot ulcer derived from non-diabetic diseases (such as occlusive vascular disease); (3) studies designed as case report, literature review, systematic review/meta analysis and letter to editors.

### Study screening

The initially retrieved records were imported into EndNote version 20.2.1 (Clarivate Analytics, Philadelphia, USA), and the duplicated studies were merged together. After then, we screened the title and abstract of each record to assess the eligibility and excluded the obviously non-related studies. The full-text of the remained studies were finally reviewed to identify the final eligible studies. The whole process of study screening was conducted by two authors independently, according to the inclusion and exclusion criteria.

### Data extraction and quality assessment

According to the PICOS principle, we perused all of the included studies, and extracted the items as follows: (1) Participants (P): patients number, drop-out patients, age, sex, body mass index (BMI), type of diabetes, lengths of diabetes history and DFU history, ulceration grade, ulceration area, glycosylated hemoglobin (HbA1C), and ankle-brachial index (ABI); (2) Interventions (I): detailed treatment protocol, perioperative management, anesthesia method, site of TTT, bone window size, fixations of external fixator and bone block, and detailed bone transportation protocol; (3) Comparisons (C): when a control group was set, the information about the treatment process was extracted; (4) Outcomes (O): healing rate, healing time, limb salvage rate, ABI, skin temperature, visual analogue scale (VAS) pain scale, and complications such as fracture at transportation site, pin-site infection, DFU recurrence; (5) Study (S): lead author’s name, publication year, country, study period, study design, and follow-up period. The process of data extraction was conducted according to the checklist of data collection which was proposed by the Cochrane Collaboration. The data were extracted by two individual reviewers independently, and cross-checked.

The quality of the RCTs, case-control studies, case series was assessed using the Cochrane Collaboration tool for assessing risk of bias, Newcastle-Ottawa scale (NOS), and JBI Meta-Analysis of Statistics, Assessment, and Review Instrument (JBI-MAStARI) scale. This process was performed by two authors independently, and the disagreement was solved by the third author.

### Statistical analysis

(1) For outcomes such as healing rate, limb salvage rate, fracture incidence at transport site, and pin-site infection risk in the TTT group, single proportional meta-analyses were performed to calculate the pooled proportions, with the “PRAW” model; (2) for outcomes such as healing time of TTT group, meta-analyses of single-group continuous data were performed to calculate the pooled mean values, with the “MRAW” model; (3) for comparisons between pre-operative and post-operative ABI, skin temperature, and VAS, meta-analyses of continuous data were conducted, with effect size of mean difference (MD); (4) for comparisons of healing rate and limb salvage rate between TTT and control groups, meta-analyses of binary data were performed with effect size of odds ratio (OR); (5) for comparison of the DFU recurrence risk between TTT and control group, meta-analyses of binary data were performed with effect size of risk ratio (RR).

The heterogeneity was tested with I^2^, and random- or fixed-effect model would be employed, when presenting with or without significant heterogeneity (I^2^>50%). Z test was used to test the statistical significance of the pooled results. Funnel plot and Egger’s/Begg’s tests (p<0.1 and p<0.05 indicate significant publication bias for Egger’s and Begg’s tests, respectively) were used to detect the risk of publication bias when five or more studies were included in a meta-analysis. If significant publication bias was detected, non-parameter trim-and-fill method was used to adjust the bias. Sensitivity analyses were performed when significant heterogeneity was evident in meta-analyses with five or more studies. The statistical significance was defined as a two-side P value of less than 0.05. The statistical procedures were completed using R 4.1.3 for Windows (R Foundation for Statistical Computing, Vienna, Austria).

## Results

### Study searching and selecting

**Figure 1:**
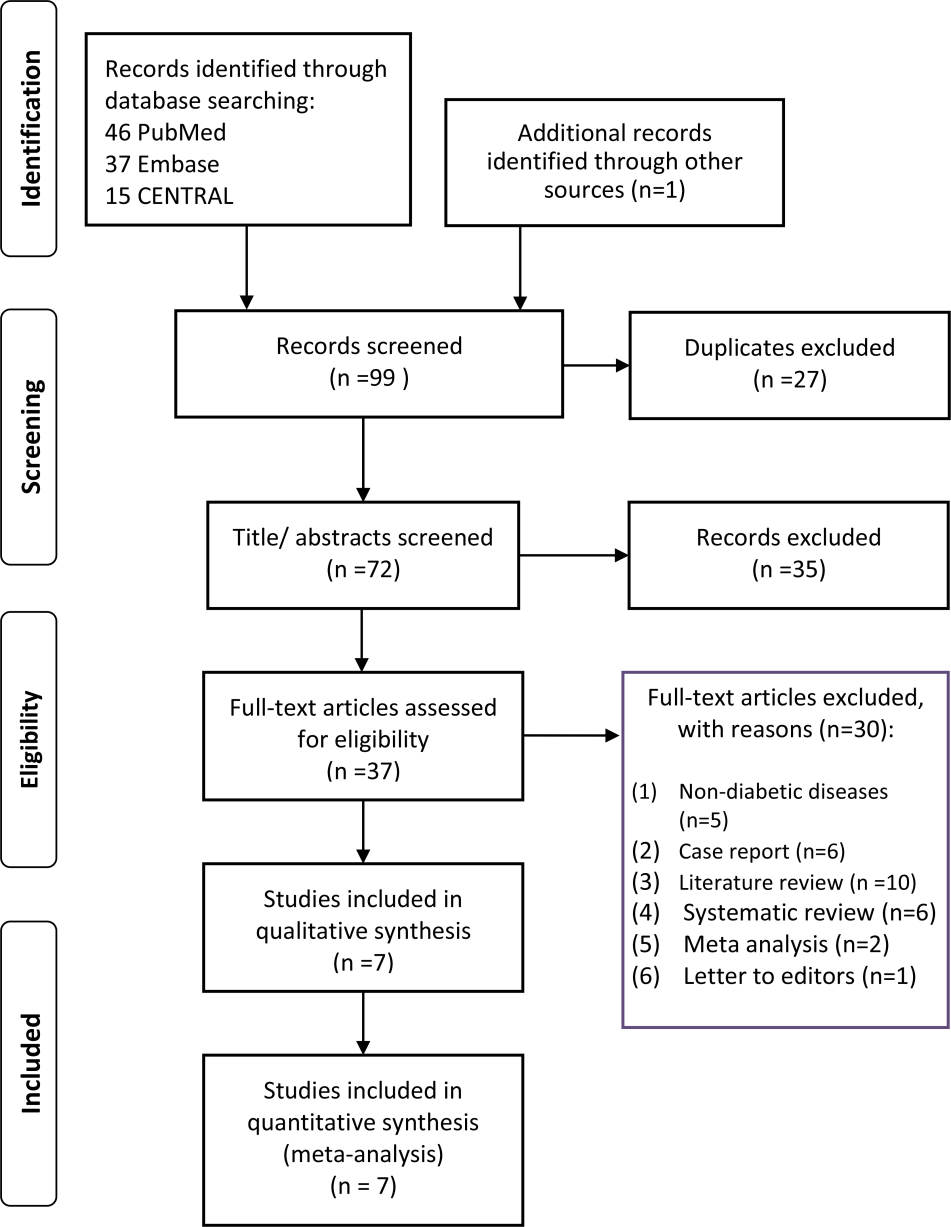
PRISMA flowchart of study searching and selecting.

The flowchart of the study searching and selecting is presented in the **Figure 1**. In total, 99 articles were retrieved through databases and manual searching. The titles/abstracts of 72 articles were reviewed after removing 27 duplicates. A total of 35 studies not related to this topic were excluded after reviewing the titles and abstracts. Then, the full-text of 37 studies were screening for final eligibility. A total of 7 studies (3, 15–20) were finally included for analysis.

### Study characteristics of the included studies

Summary of the characteristics of the eligible studies is shown in **Table 1**. All of the studies were published after 2019, in China. The study designs include single-arm RCT (n=1) (3), case-control study (n=4) (16, 18–20), and case-series study (n=2) (15, 17). A total of 818 participants were included, with 661 participants treated with TTT operation. The mean age was ranged between 40.0 ± 11.0 to 70.4 ± 6.0 years, and the male percentage was ranged between 52.6% to 83.3%. The type of diabetes was reported in 4 studies (3, 16, 17, 19), with an overall percentage of type II diabetes of 98.91% (type I: 8; type II: 727). Data about Wagner and TEXAS ulceration grades were reported in 4 (3, 15, 18, 20) and 3 (16, 17, 19) studies, respectively. In TTT and non-TTT groups, 3 and 2 patients were lost to follow-up respectively. The quality assessment result of the studies is presented in **Supplementary Table S1**.

**Table 1:** Summary of the study characteristics in the eligible studies.

Study ID	Country	Study period	Study design	Groups	N	Age	Male%	BMI	Diabetes types (I/II)	Length of diabetes	Length of DFU	Ulceration grade	Ulceration area (cm2)	HbA1C (%)	ABI	Follow-up	Drop-out
Fan ZQ, 2020 (15)	China	2015.03-2018.03	case series	TTT	30	40.0±11.0	70.0	NA	NA	NA	17.5y	Wagner 2/3/4: 8/16/6	NA	NA	NA	16.5m	0
Ding XF, 2022 (16)	China	2016.11-2019.11	case-control study	TTT_1_	115	70.4±6.0	67.8	NA	2/113	NA	3.8±0.4m	TEXAS 3D/4D: 26/89	NA	NA	0.29±0.30	12.6m	0
				TTT_2_	128	68.9±8.0	69.5	NA	2/126	NA	4.3±0.1m	TEXAS 3D/4D: 32/96	NA	NA	0.32±0.31		0
Ou SJ, 2022 (3)	China	2017.01-2019.10	single-arm Quasi-RCT	TTT	18	67.0±11.9	52.6	21.7±2.5	0/18	NA	median: 1	Wagner 4: 18	median: 2.0	7.1±0.5	NA	14.0m	1
Yuan YS, 2021 (17)	China	2016.01-2.19.10	case series	TTT	201	68.3±7.1	53.2	Median:23.5	0/201	median: 93m	NA	TEXAS 2C/2D/3D: 139/36/26	NA	Median: 10.0	NA	12m	0
Zeng ZS, 2019 (18)	China	2015.12-2017.02	case-control study	TTT	12*	55.0±7.0	83.3	NA	NA	NA	NA	Wagner 3/4:7/5	<25cm^2^:7;>25cm^2^:5	NA	NA	8w	0
				non-TTT													
Chen Y, 2019 (19)	China	2014.07-2017.03	case-control study	TTT	136	61.0±10.0	70.0	23.0±3.2	2/134	21±9y	NA	TEXAS 2B/2C/2D/3B/3C/3D: 5/7/35/6/11/72	44±10	9.7±3.7	0.37±0.06	2y	1
				non-TTT	137	60.0±11.0	64.0	23.0±3.4	2/135	20±7y	NA	TEXAS 2B/2C/2D/3B/3C/3D: 11/10/37/7/8/64	41±9	9.5±3.2	0.35±0.05		2
Fan ZQ, 2022 (20)	China	2017.03-2019.03	case-control study	TTT	21	Median: 52(42-65)	76.2	23.5±4.5	NA	median:14.5y	NA	Wagner 2/3/4: 2/14/4	NA	NA	0.45±0.13	>1y	1
				Healthy control	20	Median: 51(40-64)	75.0	23.7±6.5	–	–	–	–	–	–	–	–	0

N, patients number; TTT, Transverse Tibial Bone Transport; ABI, ankle brachial index; BMI, body mass index; HbA1C, glycosylated hemoglobin; DFU, diabetic foot ulcer; NA, not available. *a total of 12 patients were included in this study, and the operated leg was matched with the contralateral leg in analysis.

### Detailed operation process

The detailed operation process is summarized in **Table 2**. A routine debridement on the ulceration site was performed at the same time in most of the studies (15–17, 19, 20). Vacuum sealing drainage (VSD) and antibiotic bone cement (ABC) were applied at ulceration site in 2 (17, 20) and 1 (16) studies, respectively. In the peri-operative period, the managements mainly include the following four aspect: (1) antibiotics treatment according to drug sensitivity test; (2) debridement and drainage; (3) blood sugar controlling; and (4) dressing changing and disinfection. The operation was performed under general anesthesia (15, 20), nerve block anesthesia (3, 16, 17, 19), or lumbar anesthesia (15, 19, 20). The transportation site mainly located at the anteromedial area of the tibia, but the heights of the bone window were divergent among these studies. In Fan et al. (15), bone window was located at 10-20 cm below knee as they stated. In Ding et al. (16), Yuan et al. (17), and Chen et al. (19), bone window was located at the proximal tibia. In Ou et al. (3) and Zeng et al. (18), transportation site was located at middle or distal tibia. The size of bone window was reported in three studies (3, 18, 19), with different sizes. Generally, two individual pins were applied at the bone block to transport it and the tibia shaft to fixing the external fixator, respectively. All of the studies initiated the transportation at the time of 3-5 days post-operatively. Two different protocols to transport the bone block were reported: (1) 1mm per day for 14 days (3, 15, 16, 20); (2) 0.25 mm per 6h for 14 days (17, 19). Three different protocols to reset the bone block were reported: (1) 1mm per day for 14 days (15, 20); (2) 0.25 mm per 6h for 14 days (19); (3) 2 mm per day for 7 days (3, 16, 17).

**Table 2:** The detailed operation process of the included studies.

Study ID	Treatment groups	Detailed treatment protocol	Perioperative management	anesthesia method	Transportation site	Bone window size	External fixation details	Bone transportation protocol
Fan ZQ, 2020 (15)	TTT	TTT+ debridement	1.continuous closed NPD (n = 12);2.antibiotics according to drug sensitivity test;3.wound dressings changing	general anesthesiaor lumbar anesthesia	medial tibial cortex about 10 to 20 cm below knee	NA	1.Fixation: 2 half nails;2.Transportation: 2 half nails	began time: 3 to 5 days;transport: 1mm per day for 14 days;reset: 1 mm per day in the reverse direction for 14 days
Ding XF, 2022 (16)	TTT_1_	TTT+ debridement	1.emergency debridement, drainage, and foot care;2.antibiotics;3.nail passageway disinfection;4.blood sugar controlling	nerve block anaesthesia	anteromedial area of the proximal tibia	NA	1.Fixation: two 4.0 Steinmann pins;2.Transportation: two 3.0 Steinmann pins	began time: 3 days;transport: 1mm per day for 14 daysReset: 2mm per day in the reverse direction for 7days
	TTT_2_	TTT+ABC						
Ou SJ, 2022 (3)	TTT	TTT	1.blood sugar /lipids /pressure, and hypoproteinemia controlling;2.wound dressing changing and disinfection	nerve block anesthesia	anterior medial part of the middle and lower leg	7*1.8*1.5 cm	1.Fixation: two pins;2.Transportation: two pins	1.began time: 4 days;2.transport: 1mm per day for 14 days3.Reset: 2mm per day in the reverse direction for 7days
Yuan YS, 2021 (17)	TTT	mTTT+ debridement+VSD	1.IV antibiotics based on drug susceptibility testing;2.complete debridement and removing infected bone surgically, antibiotic bone cement implantation;3. continuous closed negative pressure drainage;4.blood glucose controlling;5.dressing changing and disinfection	nerve block anaesthesia	anteromedial area of the proximal tibia	NA	1.Fixation: two 4.0 Steinmann pins;2.Transportation: two 3.0 Steinmann pins	1.began time: 3 days;2.transport: 0.25 mm per 6h for 14days;3.reset: 2 mm per day for 7 days
Zeng ZS, 2019 (18)	TTT	TTT-foot	NA	NA	middle of the tibia	3.5*1.5cm (two windows)	1.Transportation: A 60*4-mm Shashi needle on each window2.Fixation: two 120*4-mm needles	NA
	non-TTT	Contralateral foot						
Chen Y, 2019 (19)	TTT	TTT+ debridement	1.antibiotics based on drug susceptibility testing;2.Standard daily wound care and off-loading casts;	spinal anesthesia or femoral nerve block	located below the tibial tuberosity	5*1.5cm	1.Fixation: two pins;2.Transportation: two pins	1.began time: 4 days;2.transport: 0.25 mm per 6h for 14 days;3.reset: 0.25 mm per 6h for 14 days
	non-TTT	standard surgical treatments*						
Fan ZQ, 2022 (20)	TTT	TTT+ debridement+VSD	1.blood glucose controlling;2.necrotic tissue debridement;3.antibiotics based on drug sensitivity testing	general or lumbar anesthesia	medial tibial cortex, approximately 10–20 cm distal to knee joint	NA	1.Fixation: two half-nails;2.Transportation: two half-nails	1.began time: 3-5 days;2.transport: 1 mm per day for 14 days;3.reset: 1 mm per day for 14 days
	Healthy control	Healthy control without any treatment	–	–	–	–	–	–

*standard surgical treatments include: debridement, revascularization, local or free flap or skin equivalent, or graft reconstruction along with negative pressure wound therapy. TTT, Transverse Tibial Bone Transport; mTTT, modified Transverse Tibial Bone Transport; NA, not available; NPD, negative-pressure drainage; ABC, antibiotics bone cement; VSD, vacuum sealing drainage.

### Results of quantitative meta-analyses

**Figure 2:**
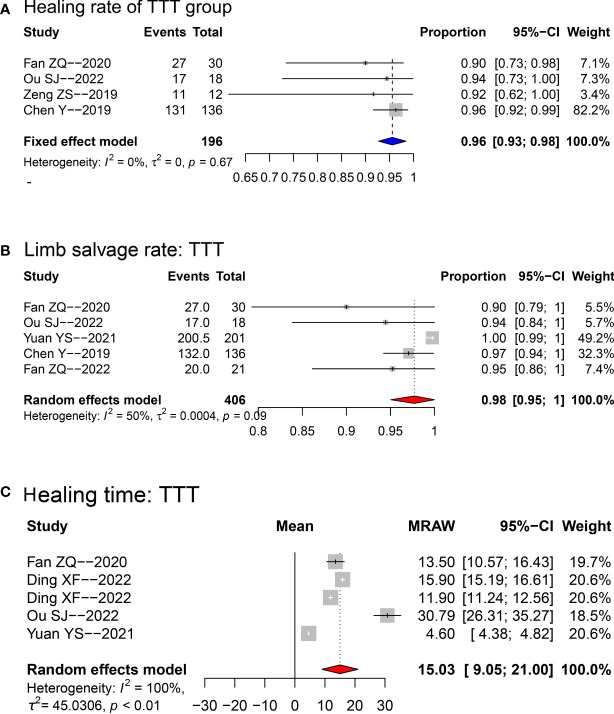
Forest plots for the meta-analyses of healing rate **(A)**, limb salvage rate **(B)** and mean healing time **(C)** in TTT group. Fixed-effect model was applied for healing rate, while random-effect model was applied for limb salvage and healing time. TTT, transverse tibia bone transportation.

**Figure 2** shows the treatment outcomes of the TTT operation at the final follow-up. The pooled healing rate was 0.96 (95% confidence interval [95%CI]: 0.93~0.98; see **Figure 2A**), using a fixed-effect model. The pooled limb salvage rate was as high as 0.98 (95%CI: 0.95~1.00; see **Figure 2B**) after treatment with TTT. The pooled mean healing time was 15.03 (95%CI: 9.05~21.00; see **Figure 2C**) months.

**Figure 3:**
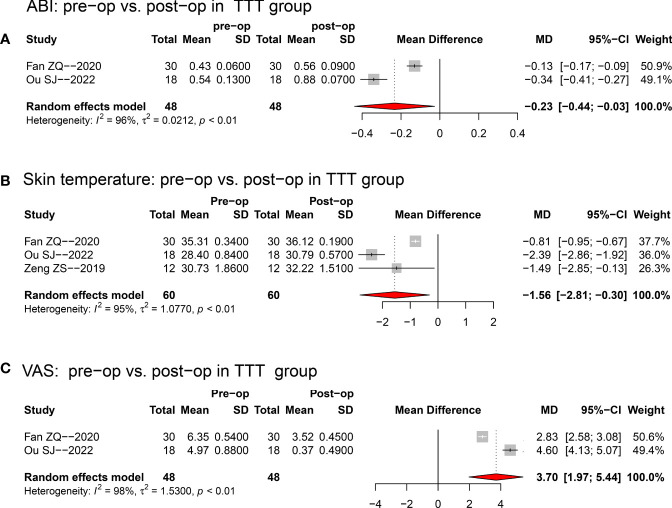
Forest plots for the comparisons between pre-operative and post-operative ABI **(A)**, skin temperature **(B)**, and VAS **(C)** in TTT group. Random-effect model was selected for the three comparisons. TTT, transverse tibia bone transportation, ABI, ankle brachial index, VAS, visual analogue scale, MD, mean difference, pre-op, pre-operative; post-op, post-operative.

When compared with the pre-operative baseline values, the ABI (random-effect model; MD: 0.23; 95%CI: 0.03~0.44; p<0.001; see **Figure 3A**), skin temperature (random-effect model; MD: 1.56; 95%CI: 0.30~2.81; p<0.001; see **Figure 3B**), and VAS-pain scale (random-effect model; MD: 3.70; 95%CI: 1.97~5.44; p<0.001; see **Figure 3C**) were all significantly improved at the final follow-up.

**Figure 4:**
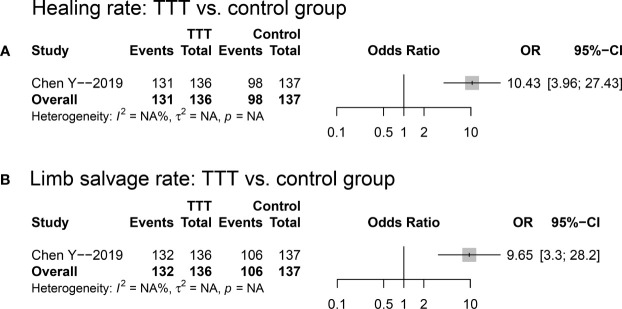
Forest plots for the comparisons of healing rate **(A)** and limb salvage rate **(B)** between TTT and non-TTT group. TTT, transverse tibia bone transportation.

When compared with non-TTT group, the TTT group was associated with higher healing rate (OR: 10.43; 95%CI: 3.96~27.43; p<0.001; see **Figure 4A**) and limb salvage rate (OR: 9.65; 95%CI: 3.30~28.20; p<0.001; see **Figure 4B**) as shown in **Figure 4**.

**Figure 5:**
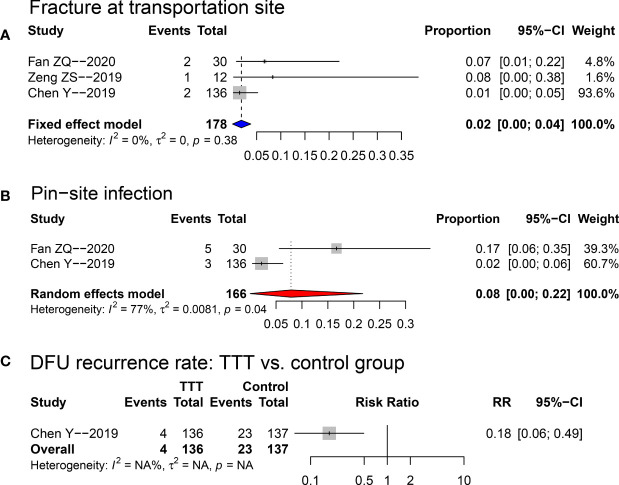
Forest plots for the risks of fracture at transportation site **(A)**, and pin-site infection **(B)** in the TTT group, and comparison of DFU recurrence rate **(C)** between TTT and non-TTT group. TTT, transverse tibia bone transportation, RR, risk ratio.

Concerning the complications of the TTT process, (1) the pooled risk of fracture at the transportation site was 0.02 (95%CI: 0.00~0.04; see **Figure 5A**); (2) the pooled pin-site infection incidence was 0.08 (95%CI: 0.00~0.22; see **Figure 5B**); (3) the DFU recurrence rate in TTT group was significantly lowered comparing to that of the non-TTT group (RR: 0.18; 95%CI: 0.06~0.49; p=0.001; see **Figure 5C**).

### Sensitivity analysis, publication bias test and trim-and-fill method

The forest plot of sensitivity analysis for healing rate in TTT group is presented in **Supplementary Figure S1**. One study (Yuan et al. (17)) was found to cause instability on the pooling result, thus it was omitted from the final pooling (see the final forest plot in **Figure 2**).

The forest plot of sensitivity analysis for limb salvage rate in TTT group is presented in **Supplementary Figure S2**. There was no study was found to cause instability on the pooling result. Significant publication bias was detected according to Egger’s (p = 0.019) and Begg’s test (p = 0.327). Thus, non-parameter trim-and-fill method was performed to adjust the bias (see **Supplementary Figure S3**), in which three studies were filled. The adjusted effect size was 0.99 (95%CI: 0.96~1.00).

The forest plot of sensitivity analysis for mean healing time in TTT group is presented in **Supplementary Figure S4**. There was no study was found to cause instability on the pooling result. No significant publication bias was detected according to Egger’s (p = 0.125) and Begg’s test (p = 0.624).

## Discussion

The main findings of the current systematic review include TTT was associated with higher healing rate and limb salvage rate when compared with control group; following operation the ABI, skin temperature, and VAS pain scale were all significantly improved; concerning the safety aspect, the TTT was associated with relatively low risks of fracture at transportation site (2%), pin-site infection (8%) and DFU recurrence (2.9%).

### The effectiveness of TTT procedure

In patients with diabetic foot, the peripheral neuropathy and vascular disease are frequently encountered, which would develop to ulceration and even amputation. The DFU, as a terminal complication of the diabetes, is of quite complex pathogenesis, being derived from a combined action of ischemia, mechanical injury, infection, and so on. Usually, the ischemia and hypoxia status is caused by damage of the small blood vessels which could not be rescued by vascular surgery (21). TTT, as a novel developed technique which was based on the Ilizarov tension-stress law, has been recently used for treatment for DFU patients with primarily satisfied success rate (18–20). These studies demonstrated that repeated mechanical stretching of the tibia bone block could stimulate the regeneration of blood vessel and accelerate the ulceration healing.

Yang et al. (22) explored the biological mechanism of the TTT procedure in rat model, and demonstrated that TTT was associated with higher blood flow in the wound area according to laser speckle imaging, and enhanced neovascularization according to double immune-labelling of CD31 and α-Smooth Muscle Actin (α-SMA). Previous studies have shown that bone distraction could enhance neovascularization through a pathway involving chemokine stromal cell-derived factor-1 (SDF-1), which is a key factor responsible for homing and migration of endothelial progenitor cells (23). In a case-control study by Chen et al. (19), they compared the treatment outcome of severe and recalcitrant DFUs with TTT and solitary standard operation, showing that tibial transverse distraction group had higher healing rate, limb salvage rate, density of small vessels, blood flow and blood volume, compared with the control group. In our results, the healing rate and limb salvage rate were demonstrated to be as high as 96% and 98% at final follow-up, and significant improvements on ABI (MD = 0.23), skin temperature (MD = 1.56) and VAS (MD = 3.70) were identified. When compared with control group, the healing rate (OR = 10.43) and limb salvage rate (OR = 9.65) were both obviously increased. These findings all confirmed the acceleration effect on neovascularization. The ABI and skin temperature is directly related with the microcirculatory perfusion of foot soft tissue. With an improved blood perfusion, sufficient oxygen and nutrition supplies can be guaranteed for ulceration healing.

However, though TTT was proven to be effective in promote ulceration healing, DFU is a multi-disciplinary condition which is difficult to be completely solved by sole TTT operation (24, 25). Many assistance procedures were applied at the same time, including debridement of the ulceration lesion (15–17, 19, 20), antibiotic bone cement filling (16), and vacuum sealing drainage (17, 20), which have all been proven to be valuable in promoting healing of ulcer wounds. At the peri-operative period, blood sugar controlling is the basic requirement to guarantee a hypoglycaemic condition for tissue repairing. Antibiotics (intravenous or per oral) according to drug sensitivity testing is also essential to control the infection and ensure the healing process. It had been reported that adequate foot care can prevent 80% of DFUs in diabetes patients (26) and effectively prevent amputation caused by ulcerations (27). Thus, it is of importance to continue standard wound care (dressing changing and disinfection) and off-loading casts in peri-operative period.

### The safety of TTT procedure

The procedure of TTT, however, is related with some potential complications, especially the fracture at the tibial bone window (15, 18, 19), infection of the pin site (15, 19) and skin necrosis at surgical site (10). Our results showed a total of 5 tibia fracture among 178 patients (pooled proportion: 2%), and 8 pin-site infections among 166 patients (pooled proportion: 8%). In Fan et al. (15), the authors suggested to avoid the fracture risk by establishing standard tibial osteotomy criteria and performing post-operative education on falling prevention. They also recommended to narrow the bone window for those patients with short stature. We firmly in favour of their proposal. Additionally, those with severe osteoporosis especially among the postmenopausal older women should be referred to standard osteoporosis treatments to prevent risk of fracture at osteotomy site. Moreover, it is of great importance to let the patients return for regular follow-up after operation. To avoid the risk of infection of pin channel, peri-operative antibiotics and daily wound care (especially pin site disinfection) are mostly important. The necrosis of local soft tissue is another major concerning during TTT, which is mainly caused by long-term continuous pressure on the skin overlying the anterior tibia (28, 29). The surgeons should try their best to preserve the blood supply of the skin flap and avoid excessive interference to the soft tissue. Post-operatively, close attention should be applied on the skin status, and termination of transportation is indicated if signs of ischemia or necrosis are evident.

In 2020, with the concerted efforts of experts in various disciplines, the “Expert Consensus on the Treatment of Diabetic Foot Ulcers Using Tibial Transverse Transport” (30) was published in China. It has emphasized the importance of further simplification of the external fixator aiming to reduce the risk of complications. During the operation period, tourniquet should not be applied, to protect the blood supply of lower limb. It is of vital significance to narrow the incision and bone window sizes and preserve the periosteum, as far as possible. Through these strategies, incidence of adverse events could be significantly reduced without addition on the difficulty of surgery process.

## Limitation

This study, nevertheless, has some limitations that must be pointed out here. Firstly, as the TTT technique was applied for DFU treatment in the most recent years, the available publications in this field are scarce with generally small sample size and retrospective design. Thus, more prospective studies with larger sample size are required in the future. Then, since the TTT procedure is predominately conducted in China, data from non-Chinese patients are not available at this stage. Thus, some further studies are required to verify the effectiveness of this operation in patients around the world.

## Conclusions

The TTT operation was demonstrated to be with high healing rate and limb salvage rate, and could significantly improve the ABI, skin temperature, and VAS after operation. When compared with the control group, TTT group provided significantly higher healing rate and limb salvage rate. However, TTT operation should be conducted with caution concerning the incidences of fracture at tibia, infection at pin channels and necrosis of skin overlying the anterior tibia.

## Data availability statement

The original contributions presented in the study are included in the article/**Supplementary Material**. Further inquiries can be directed to the corresponding authors.

## Author contributions

X-XH and Z-ZX contributed equally to this study. X-XH, Z-ZX, and G-CL: methodology, validation, formal analysis, data extraction, data curation, writing-original draft, writing-reviewing and editing, and project administration. J-YZ, L-JS, and ZC: investigation, and data processing. X-XH and HL: validation, writing-reviewing and editing. Q-FZ, and QZ: project administration. All authors contributed to the article and approved the submitted version.
