# Supplementary material for: Effectiveness of transverse tibial bone transport in treatment of diabetic foot ulcer: A systematic review and meta-analysis
Source: Front Endocrinol (Lausanne). 2023 Jan 4;13:1095361. doi: 10.3389/fendo.2022.1095361 (PMC9846025; doi:10.3389/fendo.2022.1095361)
Supplement: Supplementary file 1 [file DataSheet_1.doc]

| **Table S1. Study quality of included studies** | | | | | | | | | | | | | | | | | | | | | | |
| --- | --- | --- | --- | --- | --- | --- | --- | --- | --- | --- | --- | --- | --- | --- | --- | --- | --- | --- | --- | --- | --- | --- |
| 1. Quality assessment of case-control studies based on the Newcastle-Ottawa scale | | | | | | | | | | | | | | | | | | | | | | |
| **Study ID** | | **D_1a_** | | **D_2a_** | | | **D_3a_** | | **D_4a_** | | **D_5a_** | | **D_6a_** | | | **D_7a_** | | | **D_8a_** | | | **Total score** |
| Ding XF, 2022 [16] | | * | | * | | | * | | * | | * | | * | | | * | | |  | | | 7 |
| Zeng ZS, 2019 [18] | | * | | * | | | * | | * | | ** | | * | | | * | | | * | | | 9 |
| Chen Y, 2019 [19] | | * | | * | | | * | | * | | ** | | * | | | * | | | * | | | 9 |
| Fan ZQ, 2022 [20] | | * | | * | | | * | | * | | ** | | * | | |  | | | * | | | 8 |
| 1. Quality assessment of randomized controlled trials based on the Cochrane Collaboration tool | | | | | | | | | | | | | | | | | | | | | | |
| **Study ID** | | **D_1b_** | | | | **D_2b_** | | | **D_3b_** | | **D_4b_** | | | **D_5b_** | | | **D_6b_** | | | **D_7b_** | | |
| Ou SJ, 2022 [3] | |  | | | |  | | |  | |  | | |  | | |  | | |  | | |
| 1. Quality assessment of case series study based on the JBI-MAStARI scale | | | | | | | | | | | | | | | | | | | | | | |
| **Study ID** | **D_1c_** | | **D_2c_** | | **D_3c_** | | | **D_4c_** | | **D_5c_** | | **D_6c_** | | | **D_7c_** | | | **D_8c_** | | | **D_9c_** | |
| Fan ZQ, 2020 [15] |  | |  | |  | | |  | |  | |  | | |  | | |  | | |  | |
| Yuan YS, 2021 [17] |  | |  | |  | | |  | |  | |  | | |  | | |  | | |  | |

**Domains:** (1) D_1a_: Is the case definition adequate; D_2a_: Representativeness of the cases; D_3a_: Selection of the controls; D_4a_: Definition of controls; D_5a_: Comparability of cases and controls on the basis of the design or analysis; D_6a_: Ascertainment of exposure; D_7a_: Same method of ascertainment for cases and controls; D_8a_: Non-response rate. **(2)** D_1b_: Random sequence generation (selection bias); D_2b_: Allocation concealment (selection bias); D_3b_: Blinding of participants and personnel (performance bias); D_4b_: Blinding of outcome assessment (detection bias); D_5b_: Incomplete outcome data (attrition bias); D_6b_: Selective reporting (reporting bias); D_7b_: Other bias. low risk of bias; high risk of bias; unclear. (3) D_1c_: whether the study is based on random or quasi-random samples; D_2c_: whether the inclusion criteria are clear; D_3c_: confounding factor control; D_4c_: whether the outcome measure is objective; D_5c_: if comparison is made, whether the characteristics of each group of samples are adequately stated; D_6c_: whether the follow-up time was sufficient; D_7c_: whether the outcome of the drop-out case is stated; D_8c_: whether the outcome measure is reliable; D_9c_: whether the statistical analysis method is appropriate.
