# Supplementary material for: Effectiveness of transverse tibial bone transport in treatment of diabetic foot ulcer: A systematic review and meta-analysis
Source: Front Endocrinol (Lausanne). 2023 Jan 4;13:1095361. doi: 10.3389/fendo.2022.1095361 (PMC9846025; doi:10.3389/fendo.2022.1095361)
Supplement: Supplementary file 3 [file DataSheet_3.doc]

**Appendix 2. Searching strategies used for study retrieving**

1. **Pubmed**

#1 (((((Diabetic Foot [MeSH Terms]) OR (Foot, Diabetic)) OR (Diabetic Feet)) OR (Feet, Diabetic)) OR (Foot Ulcer, Diabetic)) OR (DFU)

#2 (((((((transverse tibial bone transport) OR (tibia transverse transport)) OR (tibial cortex transverse transport)) OR (tibial transverse transport)) OR (transversal bone transfer)) OR (bone transport)) OR (transverse tibial bone distraction)) OR (TTT)

#3 #1 AND #2

1. **Embase**

#1 'diabetic foot'/exp OR 'foot, diabetic' OR 'diabetic feet' OR 'feet, diabetic' OR 'foot ulcer, diabetic' OR 'dfu'

#2 'transverse tibial bone transport' OR 'tibia transverse transport' OR 'tibial cortex transverse transport' OR 'tibial transverse transport' OR 'transversal bone transfer' OR 'bone transport'/exp OR 'bone transport' OR 'transverse tibial bone distraction' OR 'ttt'

#3 #1 AND #2

1. **CENTRAL**

#1 MeSH descriptor: [Diabetic Foot] explode all trees

#2 Foot, Diabetic OR Diabetic Feet OR Feet, Diabetic OR Foot Ulcer, Diabetic OR DFU

#3 #1 OR #2

#4 transverse tibial bone transport OR tibia transverse transport OR tibial cortex transverse transport OR tibial transverse transport OR transversal bone transfer OR bone transport OR transverse tibial bone distraction OR TTT

#5 #3 AND #4
