# Supplementary figures and images for: Effectiveness of transverse tibial bone transport in treatment of diabetic foot ulcer: A systematic review and meta-analysis
Source: Front Endocrinol (Lausanne). 2023 Jan 4;13:1095361. doi: 10.3389/fendo.2022.1095361 (PMC9846025; doi:10.3389/fendo.2022.1095361)

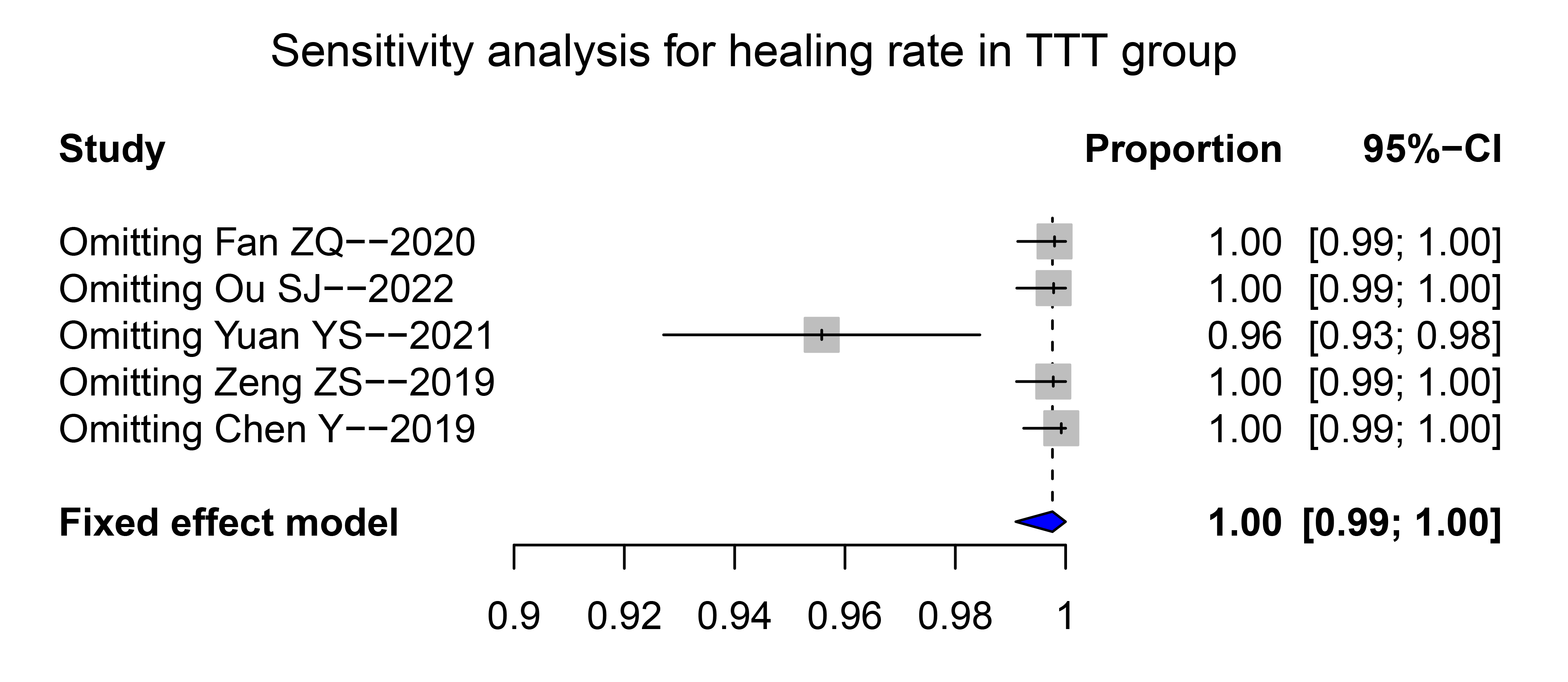

Supplement: Supplementary file 4 [file Image_1.tif]

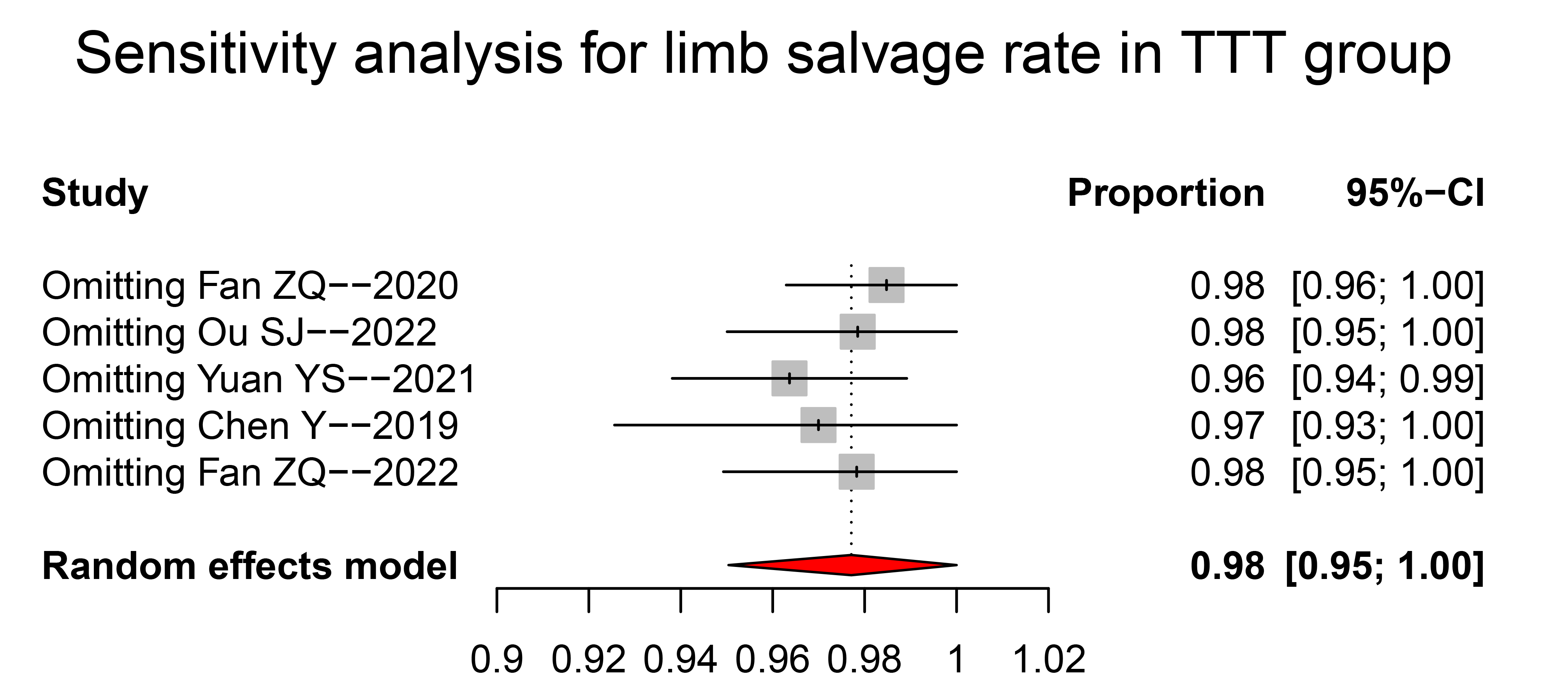

Supplement: Supplementary file 5 [file Image_2.tif]

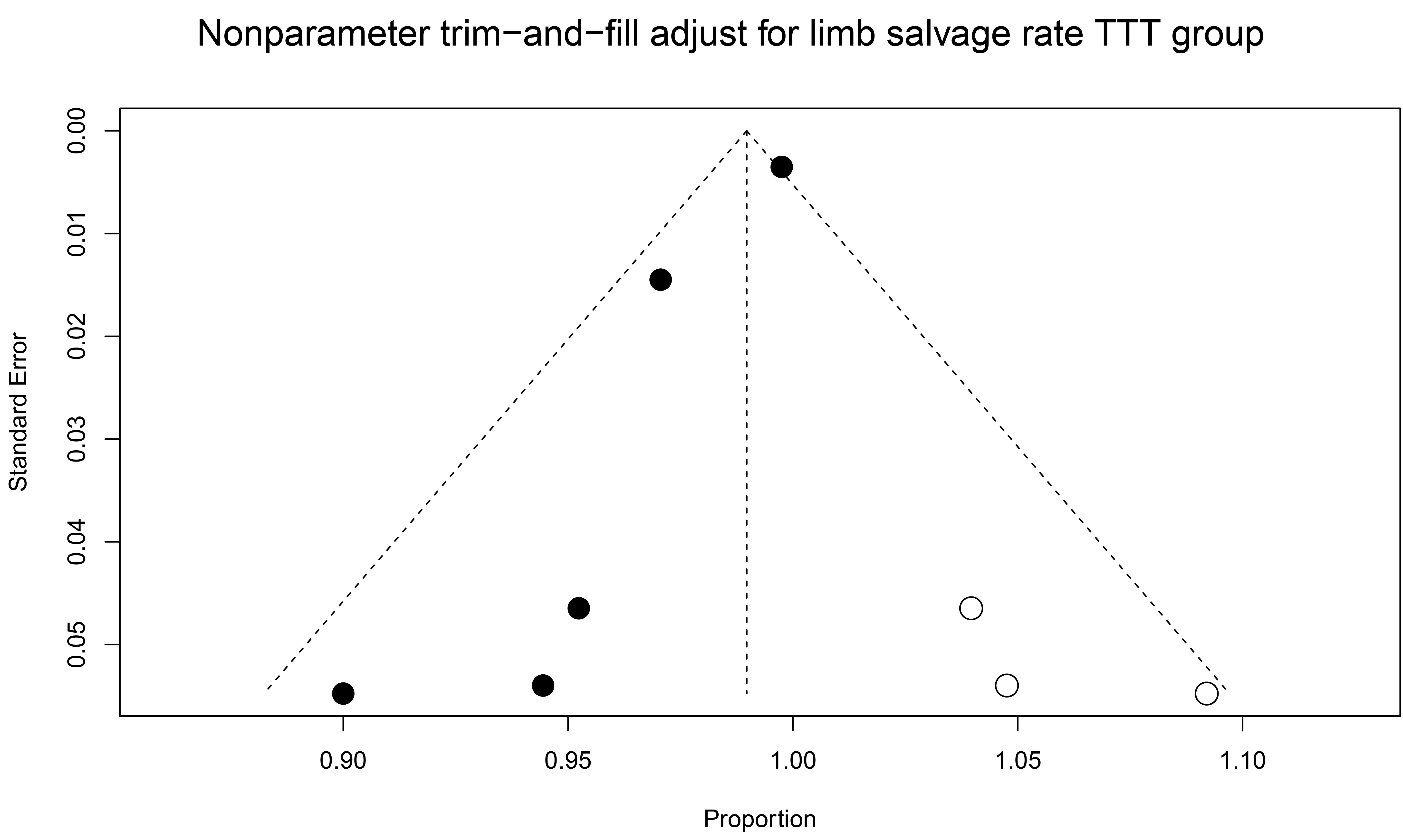

Supplement: Supplementary file 6 [file Image_3.tif]

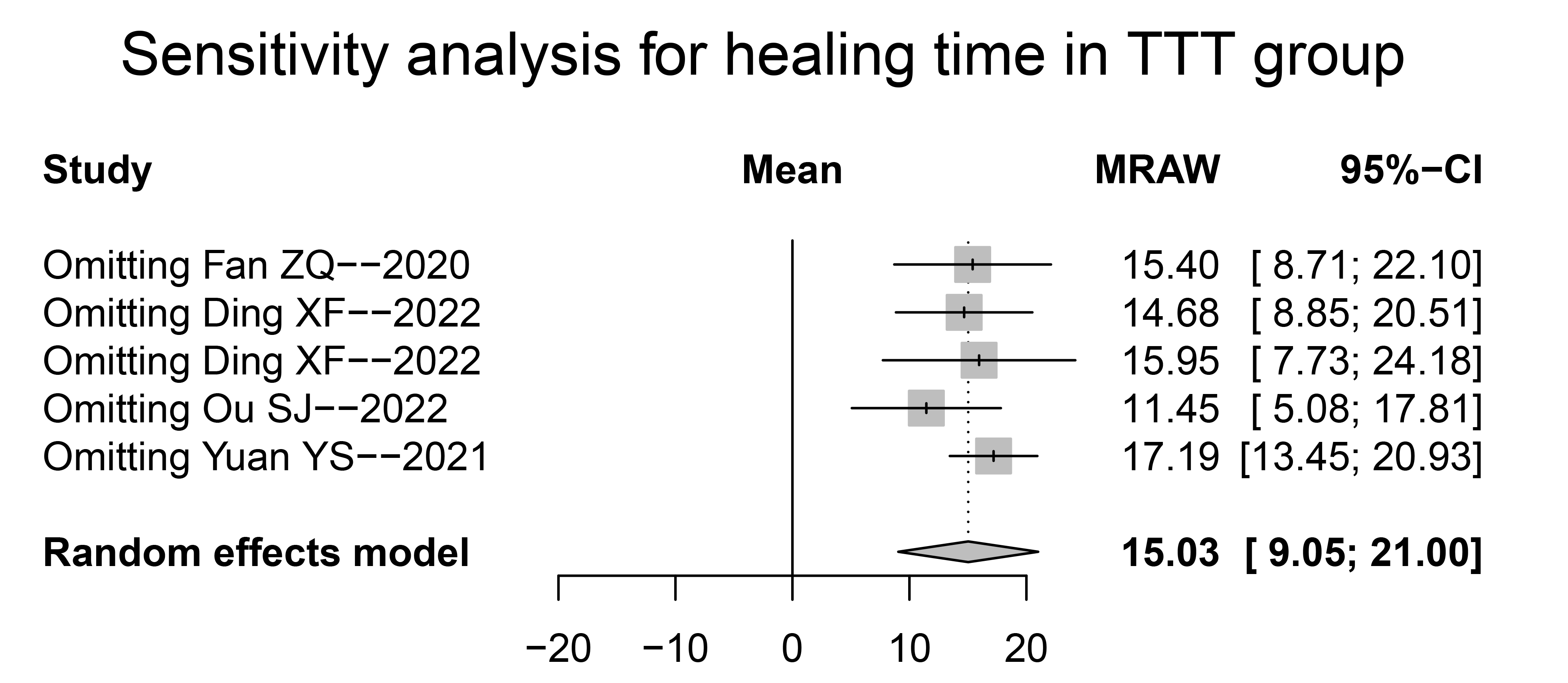

Supplement: Supplementary file 7 [file Image_4.tif]
